# Validation of the pulse decomposition analysis algorithm using central arterial blood pressure

**DOI:** 10.1186/1475-925X-13-96

**Published:** 2014-07-08

**Authors:** Martin C Baruch, Kambiz Kalantari, David W Gerdt, Charles M Adkins

**Affiliations:** 1 Empirical Technologies Corporation, PO Box 8175, 3042D Berkmar Drive, Charlottesville, Virginia 22906, USA; 2 Department of Nephrology, University of Virginia School of Medicine, Charlottesville, Virginia 22901, USA

## Abstract

**Background:**

There is a significant need for continuous noninvasive blood pressure (cNIBP) monitoring, especially for anesthetized surgery and ICU recovery. cNIBP systems could lower costs and expand the use of continuous blood pressure monitoring, lowering risk and improving outcomes.

The test system examined here is the CareTaker® and a pulse contour analysis algorithm, Pulse Decomposition Analysis (PDA). PDA’s premise is that the peripheral arterial pressure pulse is a superposition of five individual component pressure pulses that are due to the left ventricular ejection and reflections and re-reflections from only two reflection sites within the central arteries.

The hypothesis examined here is that the model’s principal parameters P2P1 and T13 can be correlated with, respectively, systolic and pulse pressures.

**Methods:**

Central arterial blood pressures of patients (38 m/25 f, mean age: 62.7 y, SD: 11.5 y, mean height: 172.3 cm, SD: 9.7 cm, mean weight: 86.8 kg, SD: 20.1 kg) undergoing cardiac catheterization were monitored using central line catheters while the PDA parameters were extracted from the arterial pulse signal obtained non-invasively using CareTaker system.

**Results:**

Qualitative validation of the model was achieved with the direct observation of the five component pressure pulses in the central arteries using central line catheters. Statistically significant correlations between P2P1 and systole and T13 and pulse pressure were established (systole: R square: 0.92 (p < 0.0001), diastole: R square: 0.78 (p < 0.0001). Bland-Altman comparisons between blood pressures obtained through the conversion of PDA parameters to blood pressures of non-invasively obtained pulse signatures with catheter-obtained blood pressures fell within the trend guidelines of the Association for the Advancement of Medical Instrumentation SP-10 standard (standard deviation: 8 mmHg(systole: 5.87 mmHg, diastole: 5.69 mmHg)).

**Conclusions:**

The results indicate that arterial blood pressure can be accurately measured and tracked noninvasively and continuously using the CareTaker system and the PDA algorithm. The results further support the physical model that all of the features of the pressure pulse envelope, whether in the central arteries or in the arterial periphery, can be explained by the interaction of the left ventricular ejection pressure pulse with two centrally located reflection sites.

## Introduction

The universal introduction of non-invasive continuous blood pressure monitors into the clinical realm remains a largely unmet challenge. While current cNIBP technologies are commonly used in research settings, there has been little penetration in intensive care or operating room settings. One indication of this status is the continuing list of publications that are related to the clinical evaluation of potential cNIBP candidate technologies [1-7].

If the accuracy of the traditional cNIBP technologies is taken as a given, which is a reasonable assumption given that the majority of devices are FDA approved and therefore meet ANSI/AAMI-SP10:2002 guidelines, there still remain significant implementation and use issues. As an example, the fact that blood pressure monitoring is largely absent in sleep lab settings strongly suggests that user comfort is one such issue [8]. Bulk, cost and associated power requirements, which essentially preclude battery usage, are others.

Pulse analysis based on hydrostatically acquired pulse signals offers a potential solution to a manageable cNIBP system. In the system used here, stable coupling to the artery can be maintained below diastole, enhancing user comfort and significantly lowering electrical power and therefore size requirements since the pump to maintain hydrostatic coupling pressure is rarely used.

At the same time there is a growing need for a truly non-invasive and easy to use blood pressure monitoring technology. Aside from the benefits provided in the ICU and OR, there are other settings where outcomes could be improved by providing expanded monitoring capability. The predictive value of night-time monitoring, particularly continuous, as opposed to blood pressure readings obtained in a clinical setting, has been recognized [9,10]. This is a highly relevant topic, as hypertension remains a problem of national importance. According to the third national health and nutrition examination survey (NHANES III) conducted from 2005 through 2008 estimated that approximately 29 to 31 percent of adults in the United States have hypertension [11]. Of similar value in the context of the increasingly speedy hospital release of patients recovering from serious interventions would be the inclusion of continuous blood pressure monitoring in the home setting. Yet current continuous blood pressure monitors are generally not suitable for the telemonitoring that these applications would require.

Blood pressure (BP) readings using conventional brachial artery measurement provide good estimates of central BP and are shown to be good predictors of cardiovascular outcomes. However, evidence suggests differential effects of BP medications on brachial artery BP readings and central aortic pressures. Monitoring central pressures may be superior to conventional BP measurements in predicting clinical outcomes after medical therapy of hypertension [12].

Similarly it is well known now that inferences about central pressure can be made based on the analysis of the pressure pulse envelope measured noninvasively in the arterial periphery, such as the radial or one of the digital arteries. As an example, the results of several recent studies support the concept that the “second systolic peak” provides the opportunity for the direct assessment of central systolic blood pressure [13-16].

The object of this work is to validate a new approach to tracking central blood pressure. The approach, based on Pulse Decomposition Analysis (PDA), [17] integrates and further develops the findings of a number of studies that have confirmed the existence of two major reflection sites in the central arteries [18,19]. The first reflection site is the juncture between thoracic and abdominal aorta, which is marked by a significant decrease in diameter and a significant change in elasticity. The reflection coefficient of this juncture is highly sensitive to blood pressure changes because of the pressure-dependent expansion of the diameter of the thoracic artery relative to that of the abdominal artery. The second site arises from the juncture between abdominal aorta and the common iliac arteries.

**Figure 1:**
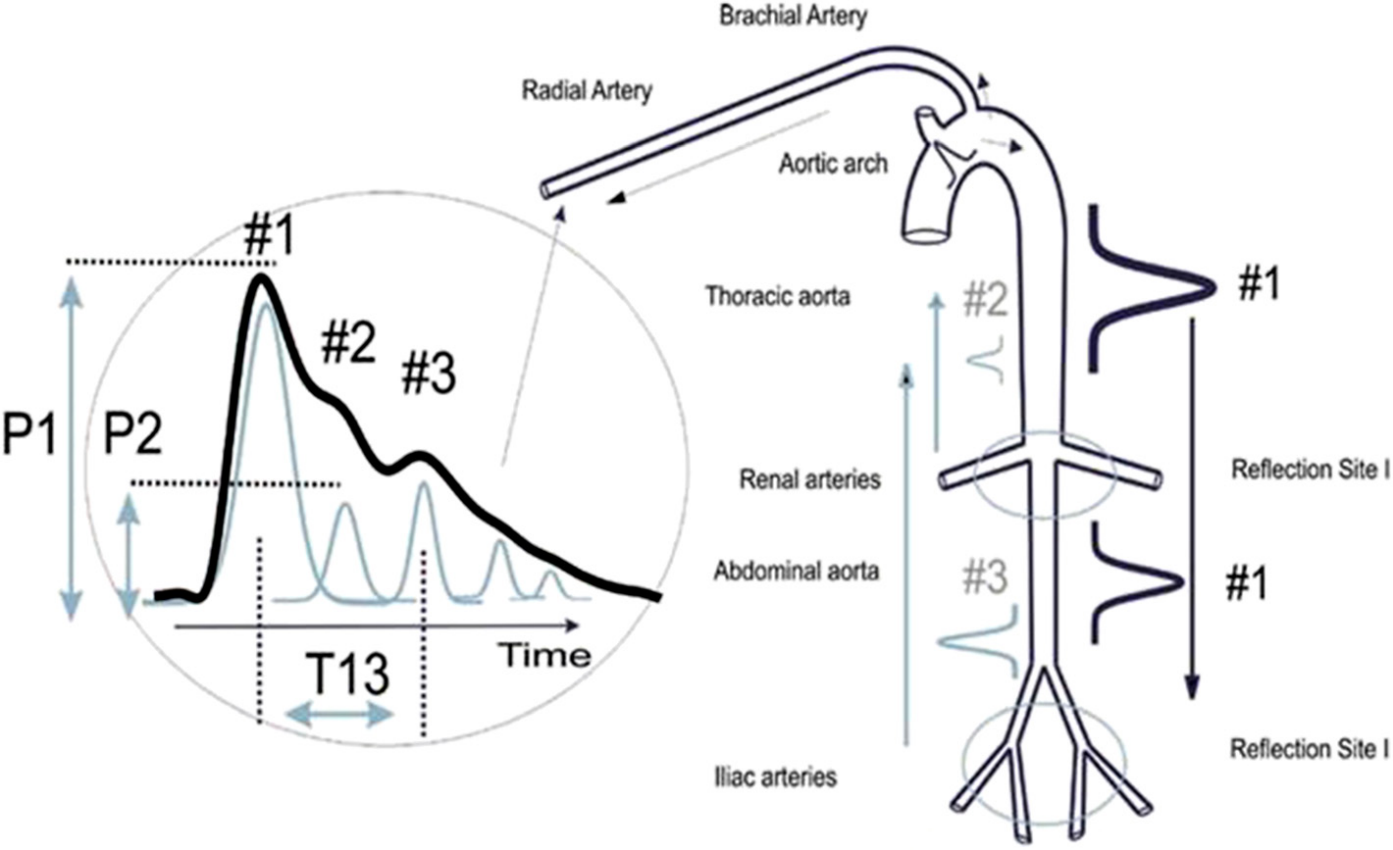
**Sketch of the aorta/arm complex arterial system and its effect on the arterial pressure pulse line shape that is observed at the radial/digital artery.** Two reflection sites, one at the height of the renal arteries, the other one in the vicinity of the iliac bifurcation, give rise to the reflected pulse (gray) that trail the primary left ventricular ejection (black).

The two reflected arterial pressure pulses are referred to as component pulses and both counter-propagate with respect to the original pulse due to the left ventricular contraction. The scenario is sketched in Figure 1. In the arterial periphery, specifically at the radial or digital arteries, these reflected pulses, the renal reflection pulse (P2), also known as the second systolic pulse) and the iliac reflection pulse (P3, also known of as the diastolic pulse because it arrives during diastole), arrive with distinct time delays. In the case of P2 the delay is typically between 70 and 140 milliseconds; in the case of P3 it is between 180 to 450 milliseconds [20].

Under optimal signal conditions as well as a sufficiently long cardiac cycle it is possible to observe 2 additional component pulses, P4 and P5, in the tail end of radial or digital arterial pulse. P4 is a re-reflection, specifically P3 re-reflecting off the renal reflection site and then re-re-reflecting off the iliac reflection site. For P5 the cycle repeats one more time. The scenario has been described by Kriz *et.al*. [21].

Quantification of physiological parameters is accomplished by extracting pertinent component pulse parameters. In the case of the beat-by-beat tracking of blood pressure the PDA model’s predictions and previous validation studies have shown that two pulse parameters are of particular importance. The ratio of the amplitude of the renal reflection pulse (P2) to that of the primary systolic pulse (P1) tracks changes in central beat-by-beat systolic pressure. The time difference between the arrival of the primary systolic (P1) pulse and the iliac reflection (P3) pulse, referred to as T13, tracks changes in arterial pulse pressure.

This paper further validates the described PDA model qualitatively as well as quantitatively through the presentation of experimental data and results obtained simultaneously from central arterial catheters and the CareTaker device, which is the hardware platform that collects the arterial pulsation data stream that is the input to the PDA formalism.

### Patients and methods

In these experiments, approved by the University of Virginia Institutional Review Board for Health Sciences Research, the aortic blood pressures of patients undergoing cardiac catheterization were monitored using central line catheters while the CareTaker system collected pulse line shapes at the proximal phalange of the pollex and an automatic cuff determined brachial blood pressure. The only criteria for exclusion were age < 18, emergency cardiac catheterization, inability to give informed consent, and a history of peripheral vascular disease. Subsequent to a preparation period of typically 10 minutes, while the patient rested in a supine position, the catheter was inserted into the femoral artery and advanced toward the heart through the aorta. As part of the study the catheter was positioned in the aorta at the height of the renal arteries for 90 seconds under fluoroscopy while the catheter signal was recorded. The CareTaker system recorded data throughout the preparation period as well as the 90 second overlap window. Both data streams were time synchronized by both matching the recording computer’s time as closely as possible to the laboratory’s central time and matching the beat-to-beat inter-beat interval variability, whose randomness provides a unique time stamped signature. PDA parameters were then extracted, beat by beat, from the non-invasively collected CareTaker data and converted to systolic and diastolic blood pressures for comparison with systolic and diastolic blood pressures obtained directly from the catheter data tracings.

The catheters/transducer system used consisted of Judkins-type catheters (6 French) and Meritrans pressure transducers manufactured by Merit Medical Systems of South Jordan, UT. The frequency response of the system ranges from 0 – 500 Hz with an accuracy of < ±1 mmHg.

### CareTaker device and PDA model

The hardware platform, which is the CareTaker device (Empirical Technologies Corporation, Charlottesville, Virginia), the model and the algorithm implementation have been described in detail elsewhere [17]. Briefly, the CareTaker is a physiological sensing system of the size of approximately a cigarette pack weighing 105 gm that communicates physiological data wirelessly via Bluetooth and whose three central physical components are a sensing pad, such as a finger cuff that couples using hydrostatic coupling, a pressure line that pneumatically telemeters the pulsations, and a custom-designed piezo-electric pressure sensor that converts the pressure pulsations, using transimpedance amplification, into a *derivative* voltage signal that is then digitized at 500 Hz, transmitted to a computer, and recorded there.

**Figure 2:**
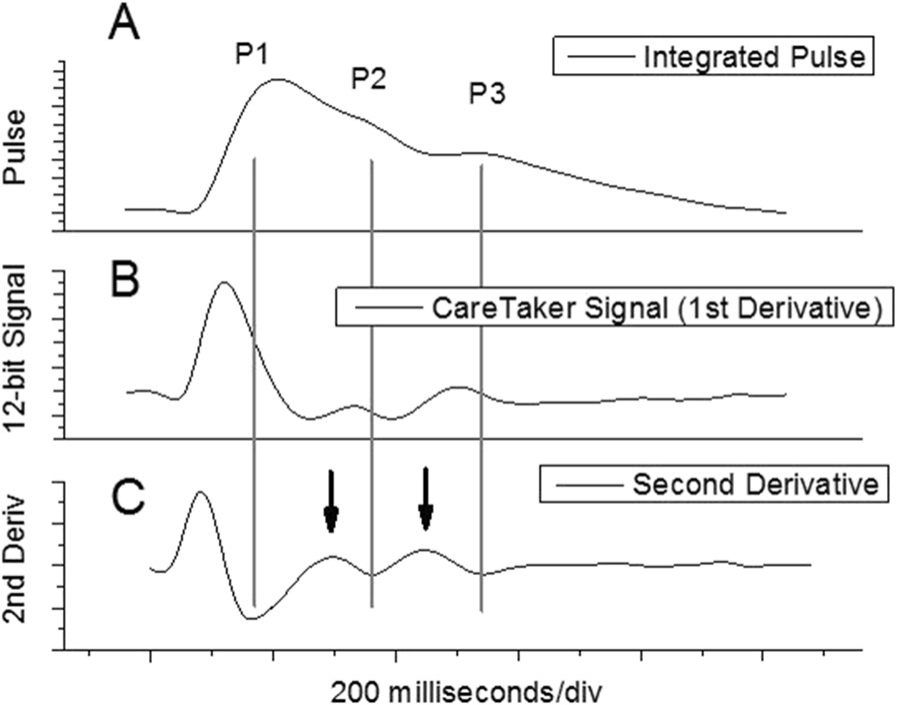
**Different pressure pulse spectra of a 21-old male athlete. (A)** Arterial pressure pulse obtained from digital integration of signal displayed in **(B)**: derivative signal of arterial pressure pulse obtained with CareTaker physiological monitor **(C)**; second derivative of arterial pressure pulse, obtained through differentiation of signal displayed in **(B)**. Component pulses are identified with, respective, P1, P2, P3. Significance of arrows is explained in the text.

The PDA model is implemented algorithmically with the following components: 1. a peak finder that identifies heartbeats in the sensor’s data stream, which is the time differentiated pulse signal, 2. a differentiator that produces the second derivative of the detected heartbeat, which is then used to find the locations of the component pulses and 3. a digital integrator, implemented as a Bessel filter, that generates the integrated pulse wave form from the *derivative* raw signal stream, and from which relative component pulse amplitudes are determined. The different spectra are presented in Figure 2, which displays respectively for a detected heartbeat, the digitally integrated pulse shape (A), the raw signal of the detected heartbeat (B), and the derivative of the raw signal version (C), i.e. the second derivative of the pulse signal.

The detection of all three component pulses is accomplished in the second derivative spectrum of the pulse (Figure 2, graph C). Component pulses P2 and P3 are detected using simple peak detection. However, since the second derivative pulse shape is inverted relative to a given original pulse shape, it is the corresponding *minima* that represent the locations of P2 and P3 (long vertical gray lines). To facilitate detection the *maxima* preceding the minima are detected first (black vertical arrows in the second derivative spectrum), and then the subsequent signal minima. To avoid detection of spurious noise spikes, the peak detector operates on data smoothed over 20 data points, corresponding to 40 milliseconds. Since the previously stated physiologically plausible arrival time windows of P2 and P3 relative to the onset of the pulse envelope are known, a decision-making loop is used to down-select peaks in the event that additional peaks are detected.

**Figure 3:**
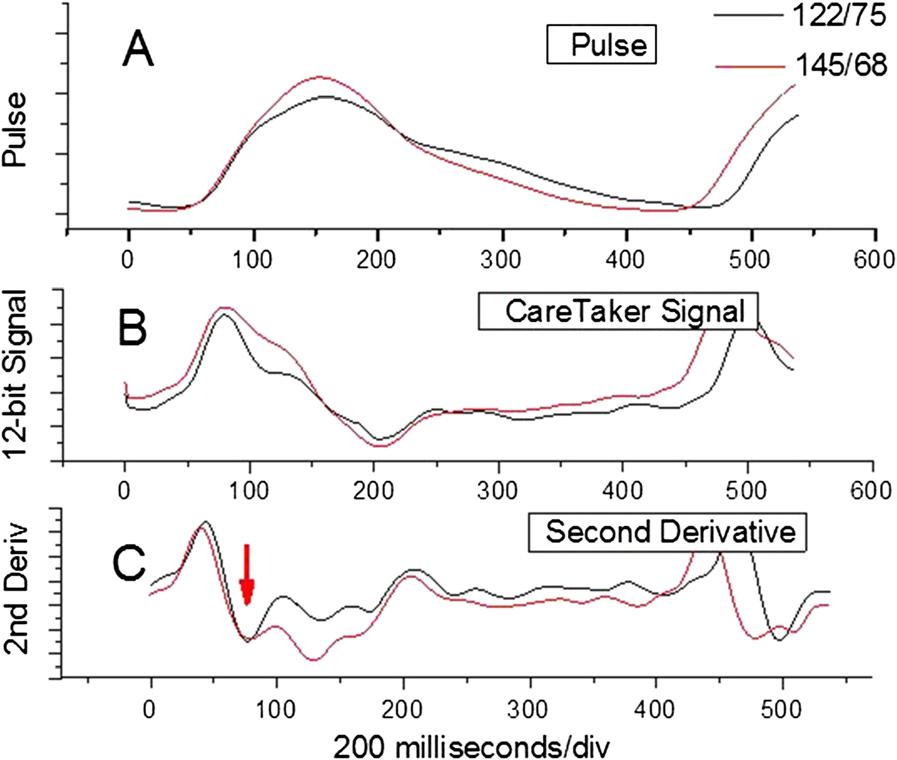
**Different pressure pulse spectra of a 65-old male at two different blood pressures. (A)** Arterial pressure pulses obtained from digital integration of signal displayed in **(B)**: derivative signals of arterial pressure pulse obtained with CareTaker physiological monitor **(C)**; second derivatives of arterial pressure pulse, obtained through differentiation of signal displayed in **(B)**. Note the merging of P2 and P1 in the pulse envelopes **(A)** as well as the increase in the shoulder between data points 100 and 150 in the derivative signals **(B)**. Significance of red arrow is explained in the text.

The detection of P1 is also accomplished in the second derivative spectrum, but requires sometimes more sophistication because P1 is not an isolated peak in situations where the P2 amplitude is large and both peaks overlap significantly. This can either be due to a significant increase in systole or due to a persistently high renal reflection, commonly referred to as a high “augmentation index”. An example of a pressure-induced enlargement of the P2 amplitude is shown in Figure 3 and presents the pressure pulse envelope collected 10 minutes apart at the indicated blood pressures. In this higher-pressure situation P1 is distinguishable by at least a shoulder. In high “augmentation index” and/or high systole cases this peak can become indistinguishable in the pulse spectrum as P2 effectively merges with P1, a condition shown and remarked on by other researchers [22]. In this case the first positive-going inversion in the second derivative spectrum (Figure 3C) after the pulse onset, indicated by a red arrow, indicates the position of the P1 component pulse. Once the temporal locations of the component pulses have been determined, the T13 interval, the time delay between systolic (P1) and iliac peak (P3), is calculated.

The P2 to P1 amplitude ratios are calculated from the integrated pulse spectrum (Figure 2A). In regard to P1, this amplitude is determined *on the pulse envelope*, but at the temporal location found, as described above, in the second derivative spectrum (Figure 2C). The P2 amplitude is obtained by calculating the integrated average amplitude over a 50 millisecond centered on the temporal location of P2. This approach was found to be more robust in light of the dynamic response of P2, with regard to temporal as well as amplitude shifts, to blood pressure variations.

### Statistical analysis

We present regression coefficients and linear fits between central catheter blood pressures and blood pressures obtained from the conversion of the PDA parameters P2P1 and T13 obtained from the non-invasively obtained arterial pulse data. Individual patient data, histogram distributions of the slopes of the linear fits for individual patients, as well as overall linear fit-based comparisons between central catheter blood pressures and blood pressures obtained from the PDA parameters using a single set of conversion constants are presented. Bland-Altman comparisons of the two sets of blood pressures are also provided.

## Results

Table 1 summarizes the patient data and the indications of catheterization. A total of 63 patients were included in the study and one data run per patient was collected. The principal indications for catheterization were coronary artery disease (28.5%), chest pain (28.5%), dyspnea (15.8%), and transplant-related evaluations (11%). Data from 4 patients could not be analyzed. In one case, the central line catheter failed and no data was recorded. In another case no overlap of the inter-beat interval could be obtained because the matching overlap section in the catheter data was lost due to a data storage error. In two cases the CareTaker system failed to acquire data; in one case due to a low battery condition, in the other due to a disconnected hose connecting the CareTaker sensor housing to the finger cuff. Remedying any patient-side and CareTaker-related failure during the catheterization procedure was not an option during the medical procedure. For the remaining patients all 90 seconds of data were analyzed.

**Table 1:** Summary of patient data and indications of catheterization

**Patient information**	
Age	62.7 ± 11.5
Gender	38 m/25 f
Height (cm)	172.3 ± 9.7
Weight (kg)	86.8 ± 20.1
Mean arterial pressure (mmHg)	91.3 ± 13.4
heart rate (beats/min)	67.9 ± 13.8
Indication for catheterization	Patients in category
Mitral regurgitation	3
Chronic obstructive pulmonary disease	5
Coronary artery disease	18
Transplant-related evaluation	7
Coronary artery bypass graft	5
Aneurysm repair-related assessment	2
Aortic stenosis	3
Chest pain	18
myocardial infarction	4
dyspnea	10
Raynaud phenomenon	1
Ventricular fibrillation/cardiac arrest	1
Atrial fibrillation	1
Pacemaker change-related	1

### Comparisons of overlap episodes of PDA parameters and central blood pressures

Overlaps of the CareTaker data streams and the central catheter data streams were finalized, after an initial alignment based on data collection systems clocks, by matching inter-beat interval spectra obtained via pulse detection from both data streams. In addition singular events, such as a skipped heartbeat, were used to establish overlap, when available.

**Figure 4:**
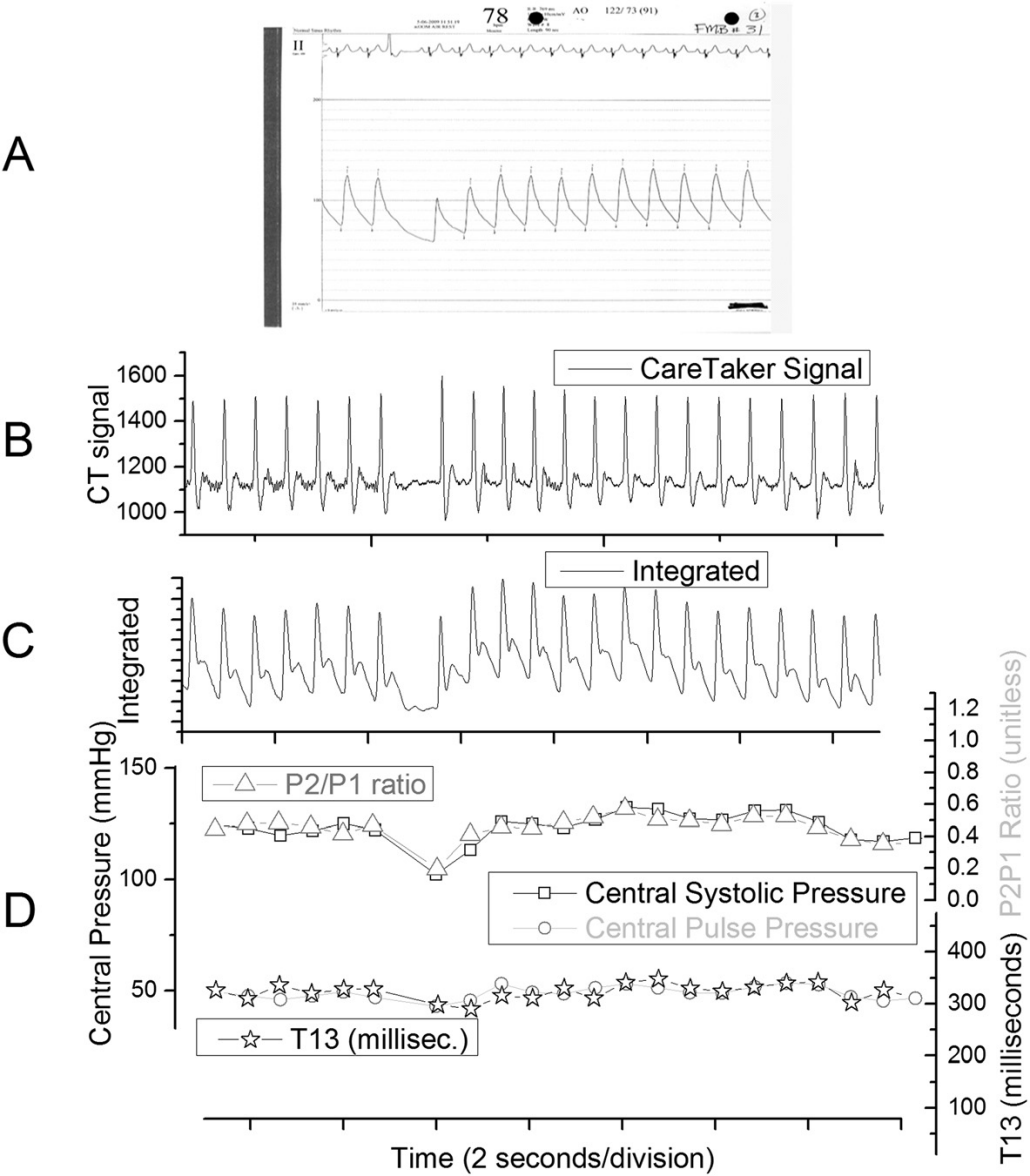
**Overlap of raw and derived signals for patient 31. (A)** Printout of the central line blood pressure data; **(B)** derivative signal of arterial pressure pulse obtained with CareTaker physiological monitor; **(C)** arterial pressure pulse obtained from digital integration of signal displayed in **(B)**; **(D)** overlay of A-line systole, A-line pulse pressure, P2P1 ratio and T13 time interval.

Figure 4 presents an example of an overlap of the raw and derived signals for patient 31. The top graph, 4A, presents the printout of the central line blood pressure data, while 4B and 4C display, respectively, the raw CareTaker signal and the digitally integrated signal. Graph 4D displays an overlay of the relevant extracted parameters, specifically A-line systole, A-line pulse pressure, the P2P1 ratio and the T13 time interval, both derived from the CareTaker signal as described.

**Figure 5:**
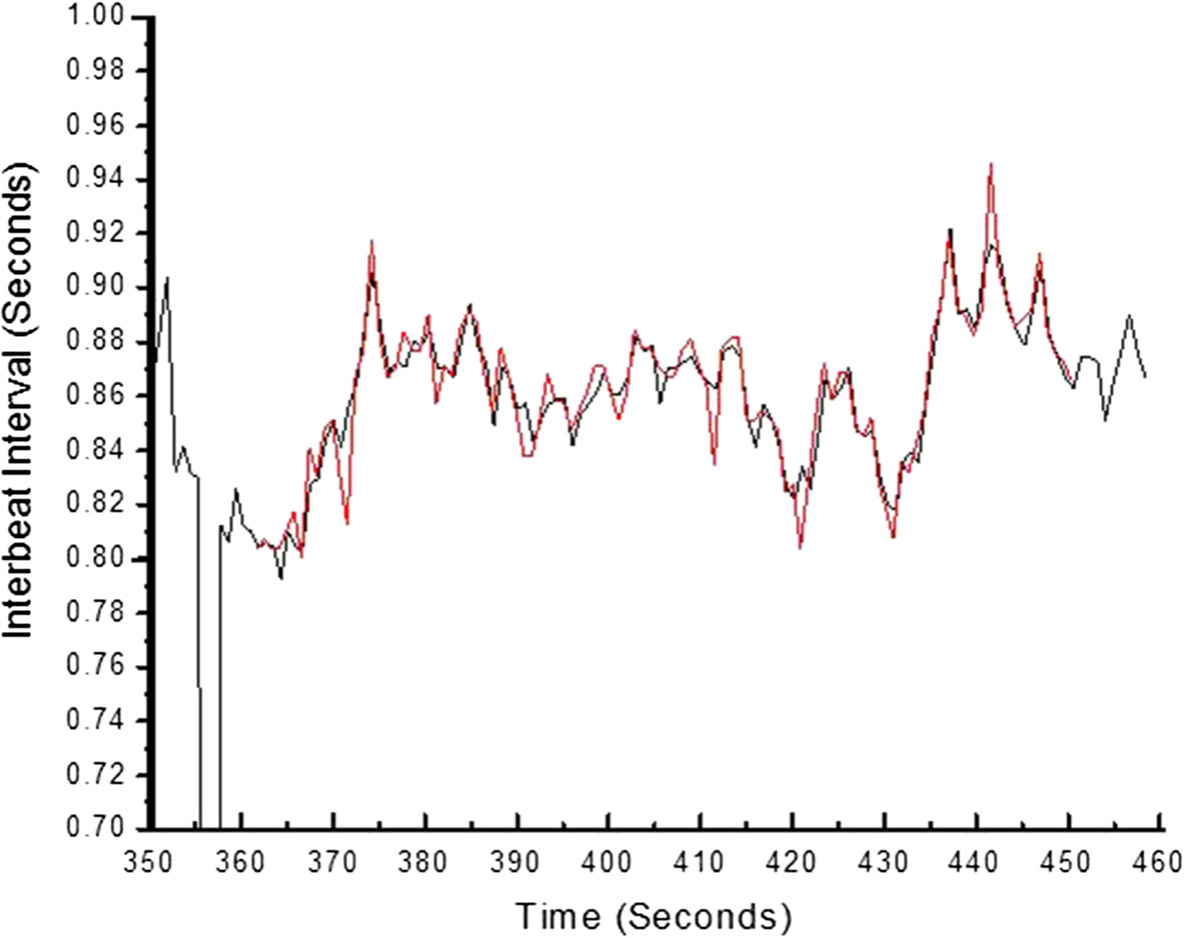
Overlap of the inter-beat interval series obtained from central line catheter (red) and data obtained with the CareTaker physiological monitor (black) for patient 38.

**Figure 6:**
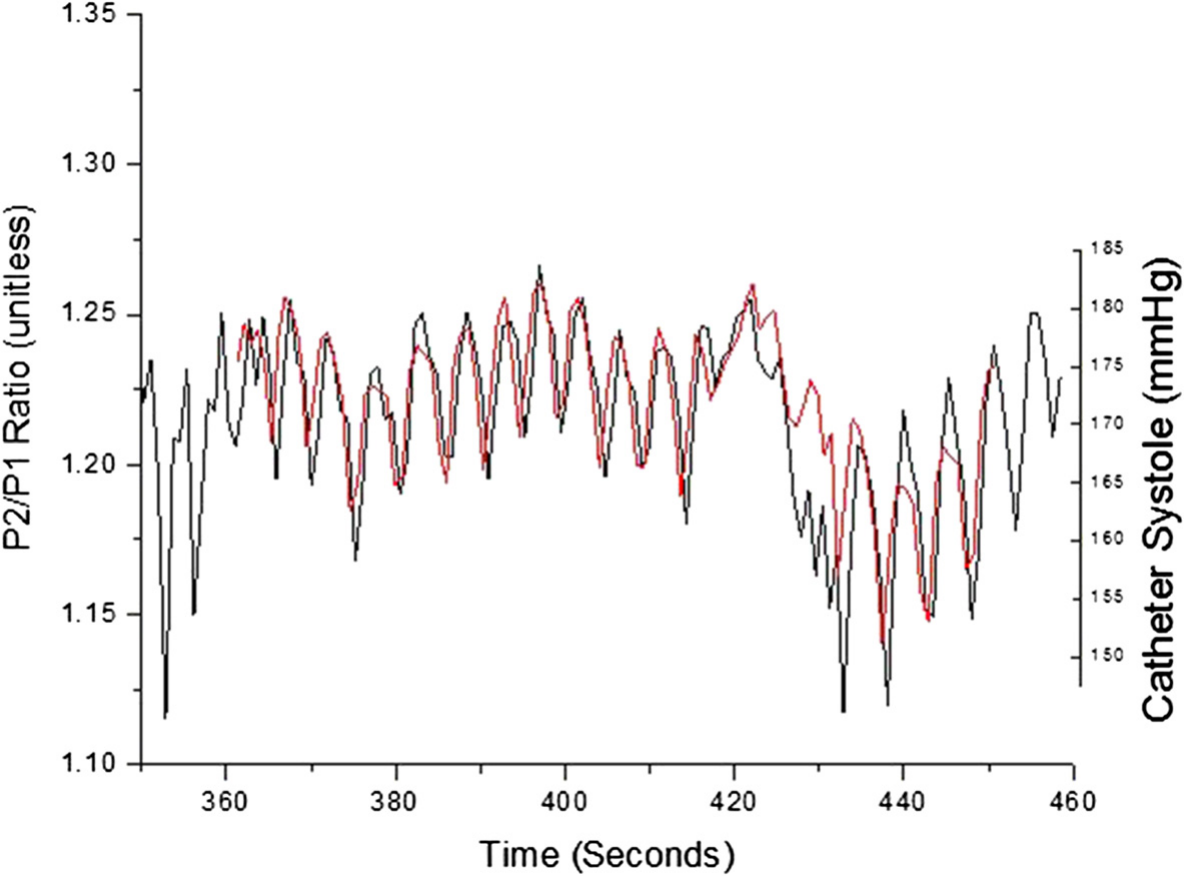
Overlap of central systolic pressure (red) obtained from catheter signal and P2P1 ratio obtained from PDA analysis of non-invasively obtained arterial signal (black) for patient 38.

**Figure 7:**
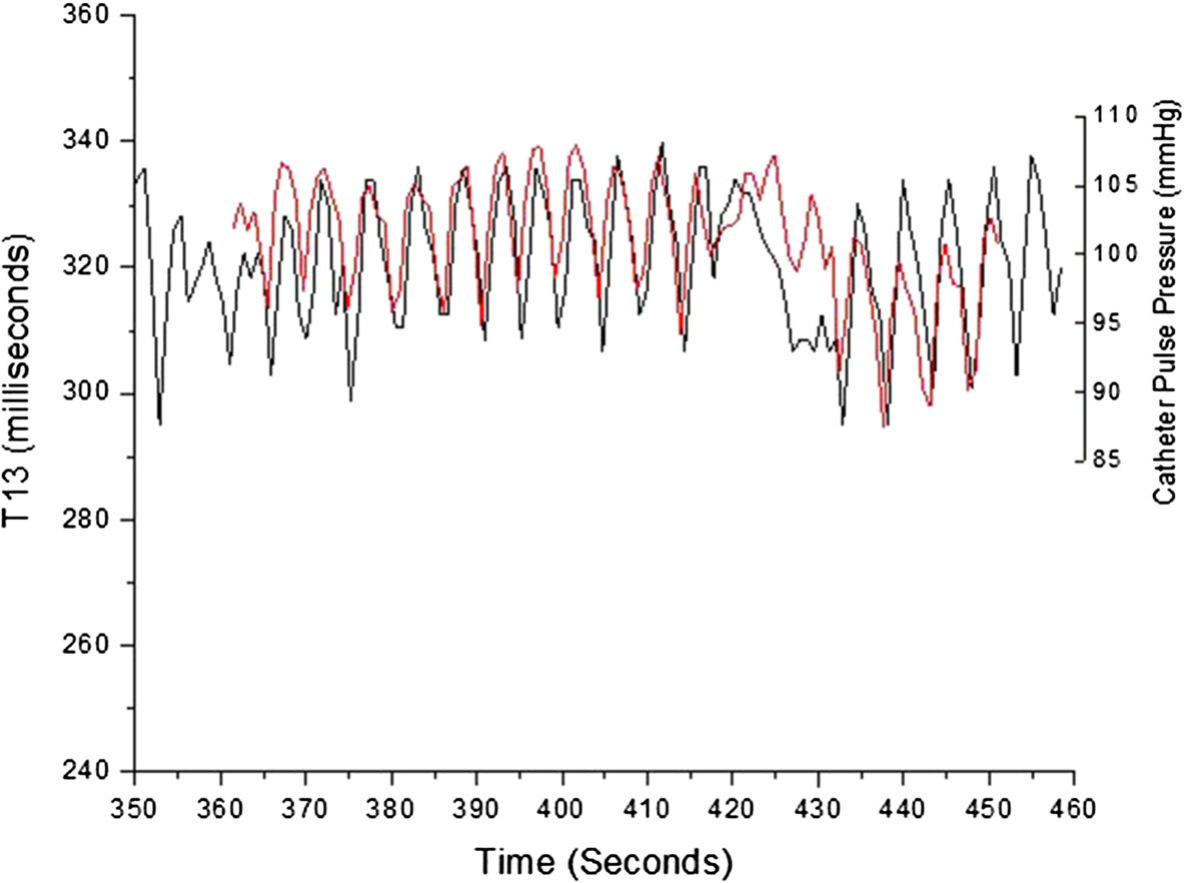
Overlap of central pulse pressure (red) obtained from catheter signal and T13 delay time obtained from PDA analysis of non-invasively obtained arterial signal (black) for patient 38.

**Figure 8:**
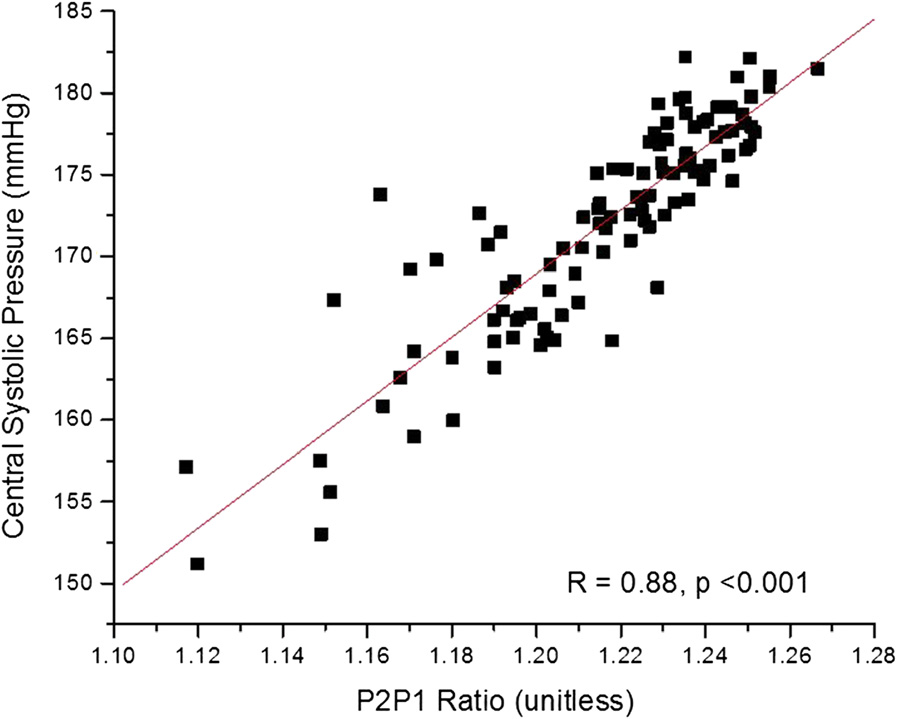
**Correlation of P2P1 parameter with central line systolic pressure, as shown in Figure **5**, for patient 38.**

**Figure 9:**
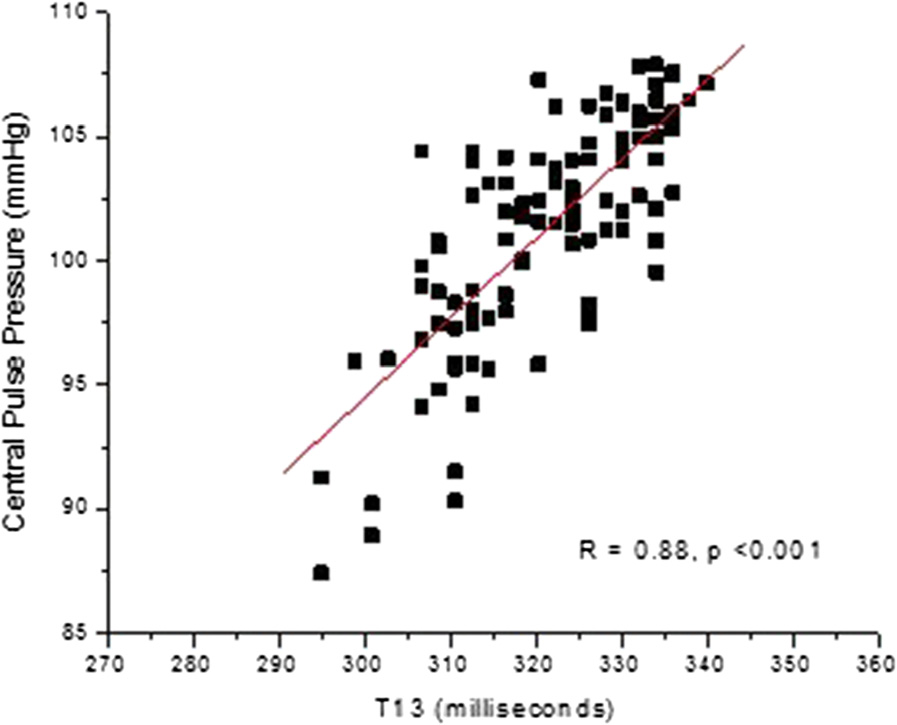
**Correlation of T13 parameter with central line pulse pressure, as shown if Figure **7**, for patient 38.**

Figures 5, 6 and 7 present an example of such an overlap of extracted parameters over the entire 90 second scan length for patient 38, a former smoker with coronary artery bypass graft (CABG) surgery three years prior. Specifically, Figures 5, 6 and 7 display the relative overlap of, respectively, inter-beat interval, central systolic blood pressure and the P2P1 parameter, as well as central pulse pressure and T13. Figures 8 and 9 display the corresponding correlations of the PDA parameters and central pulse blood pressures, for patient 38.

For patient 38, the linear model used to perform the conversion of the P2P1 parameter to a systolic blood pressure was (100 × P2P1 (unit less ratio) + 0.945), while the corresponding linear conversion for the T13 parameter to pulse pressure was (0.2 × T13 (milliseconds) + 0.0025). The slope factors of each equation for this patient are the same slope factors that were used for the conversions for all patients, and were obtained through an overall linear fit analysis of the entire patient data set. In contrast, the offset factors of the conversion equations are patient-specific and were obtained by chi square minimization of the fit of both data streams.

### Overall results

**Figure 10:**
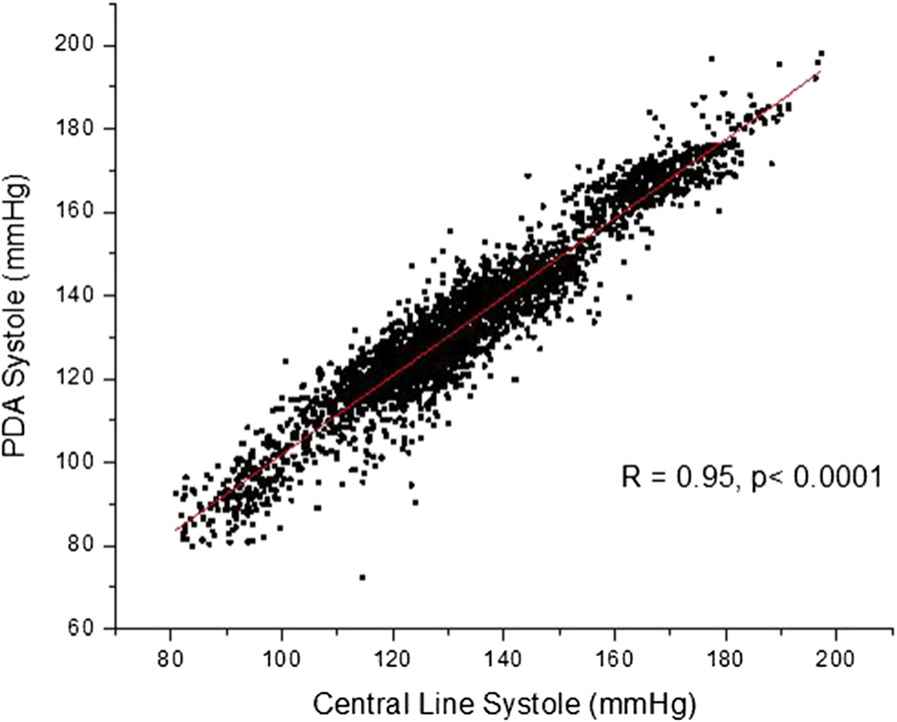
Overall correlation of systolic blood pressures obtained through conversion of PDA parameters from non-invasively obtained arterial pulse signal, and central systole.

**Figure 11:**
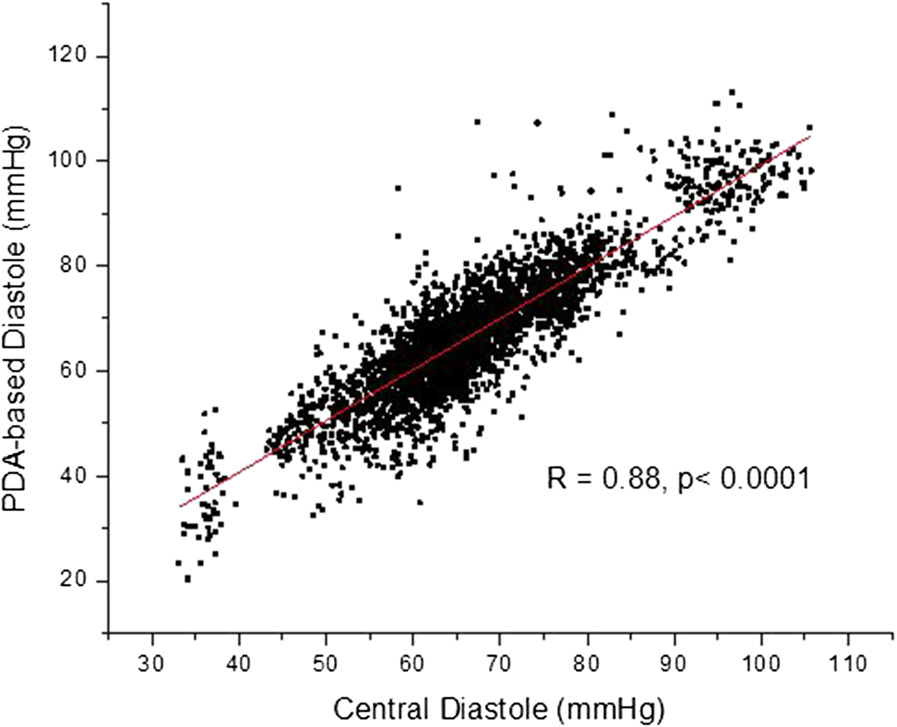
Overall correlation of diastolic blood pressures obtained through conversion of PDA parameters from non-invasively obtained arterial pulse signal, and central diastole.

**Figure 12:**
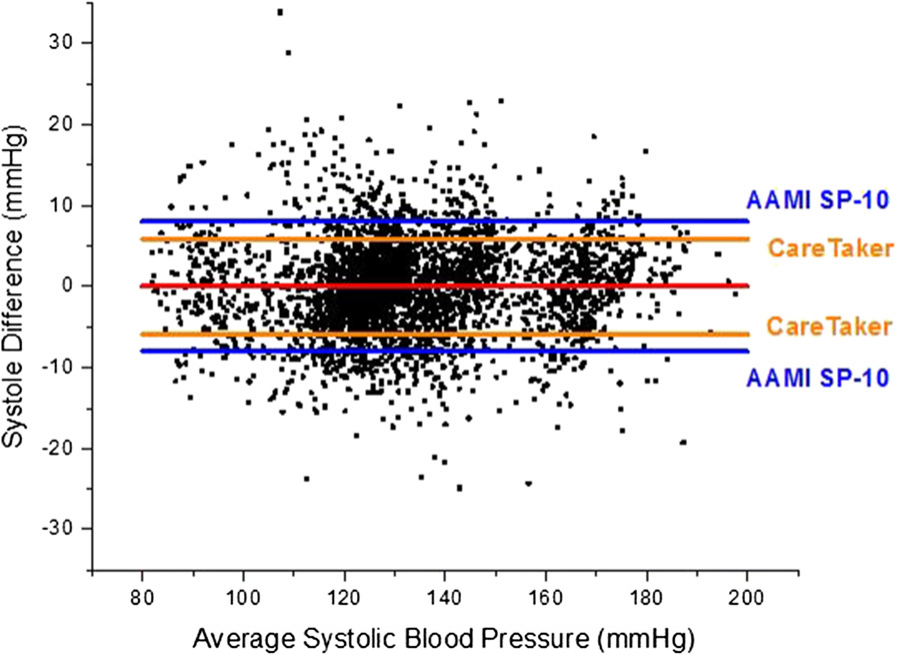
**Bland-Altman comparison of systolic pressure difference versus systolic pressure average.** Standard deviation: 5.87 mmHg.

**Figure 13:**
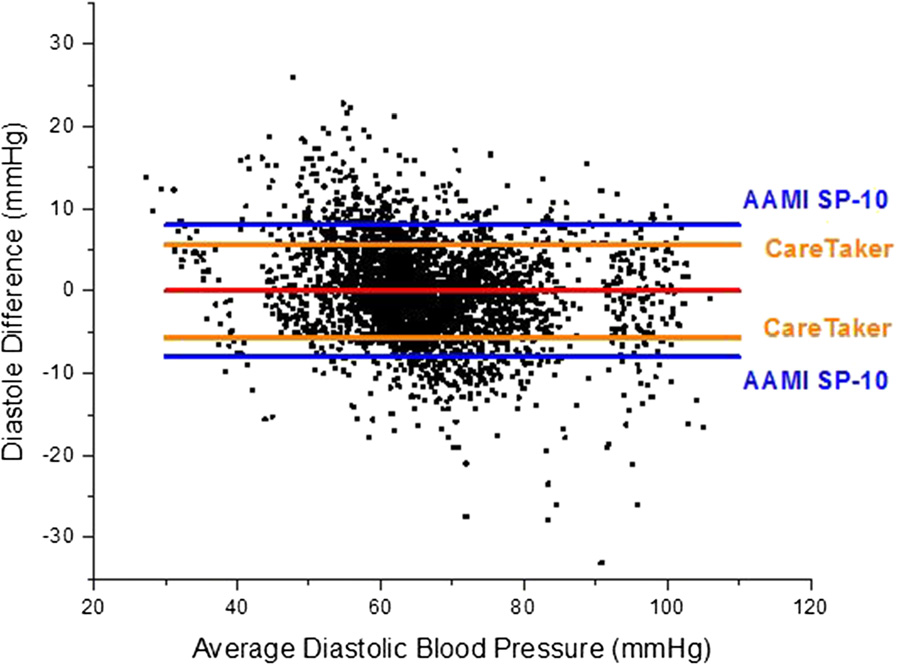
**Bland-Altman comparison of diastolic pressure difference versus diastolic pressure average.** Standard deviation: 5.69 mmHg.

The overall results of the study are presented as blood pressure correlations and Bland-Altman graphs in Figures 10, 11, 12 and 13. The correlations were established by converting the PDA parameters P2P1 and T13 into systolic and diastolic blood pressures, respectively, for each patient. For the gains the previously mentioned values of 100 and 0.2 were used throughout. The linear offset for each patient was obtained from linear fits. The Bland-Altman graphs display the same data in the format of difference as a function of the average of paired readings. The correlations were statically significant (PDA systole/central systole: R square: 0.92 (p < 0.0001), PDA diastole/central diastole: R square: 0.78 (p < 0.0001). The Bland-Altman comparisons yielded standard deviations within the Association for the Advancement of Medical Instrumentation (AAMI) SP-10 limits of 8 mm Hg (PDA systole/central systole: 5.87 mmHg (p < 0.0035), PDA diastole/central diastole: 5.69 mmHg (p < 0.001).

### Pulse decomposition analysis (PDA) conversion factors

**Figure 14:**
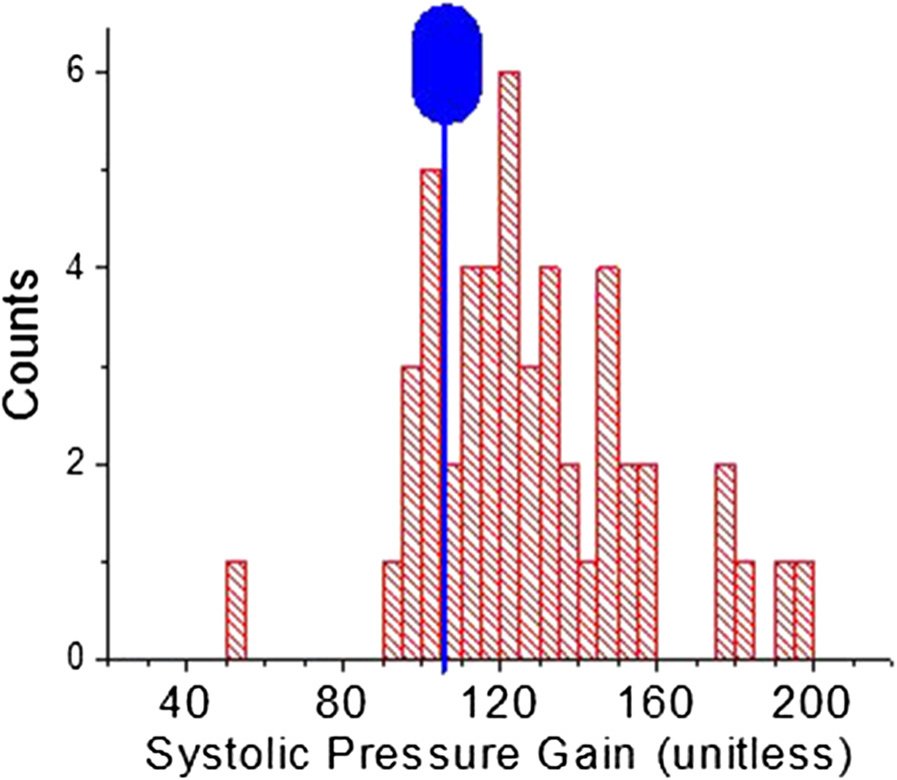
**Histogram distribution of the slope parameters of the linear conversions from P2P1 to systole.** Blue marker is described in the text.

**Figure 15:**
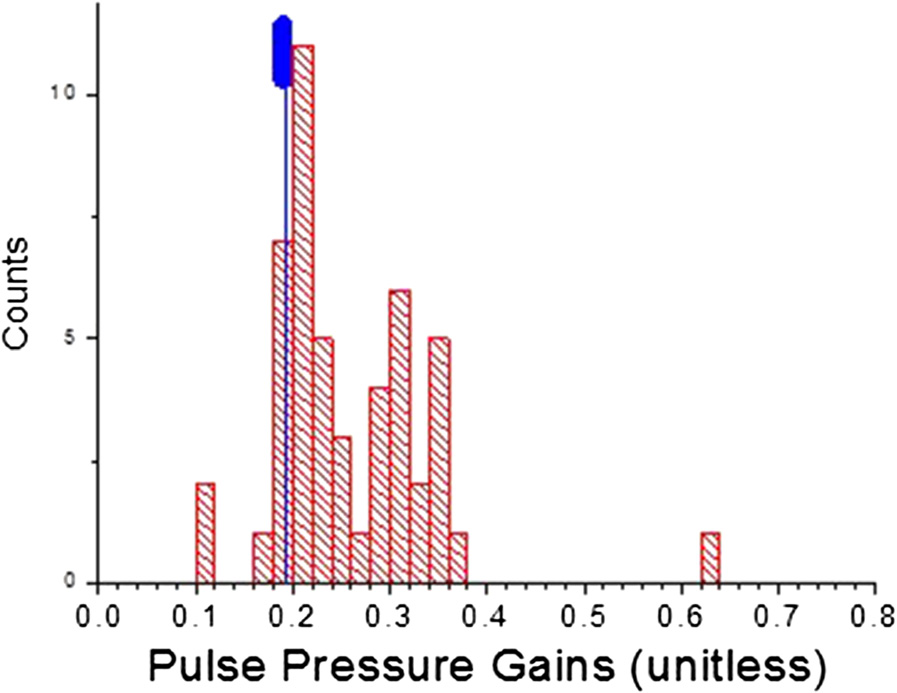
**Histogram distribution of the slope parameters of the linear conversions from T13 to pulse pressure.** Blue marker is described in the text.

Figures 14 and 15 present, respectively, histogram distribution graphs of the slopes of the correlations, for each patient, for P2P1 and systolic blood pressures, and T13 and diastolic blood pressures, respectively. The distributions’ peak amplitudes are close to the slope factors obtained from previous studies. Of note is the fact that the distributions’ tails extend to the higher-gain side for both factors, indicating that for these patients smaller changes in arterial distension (P2P1) and pulse travel time (T13) are associated with larger changes in blood pressure.

### Qualitative data assessments

**Figure 16:**
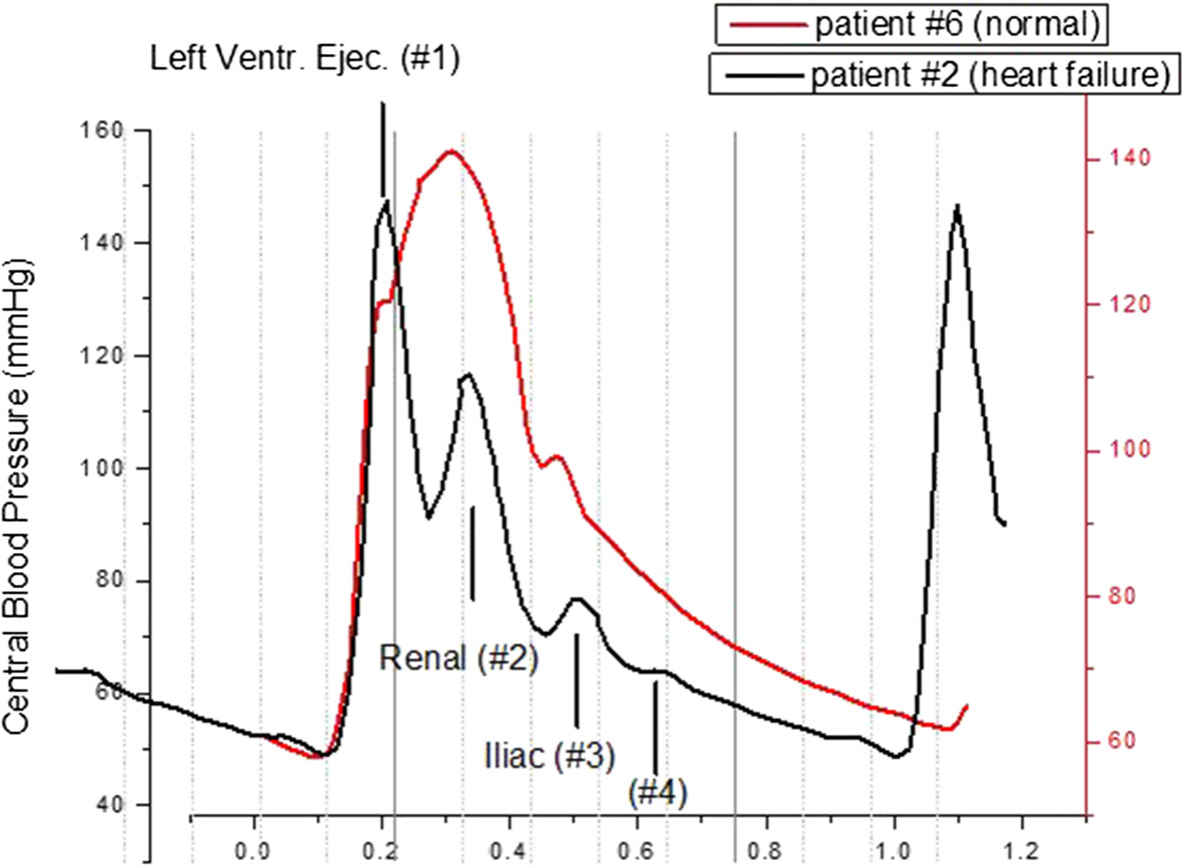
**Overlap of the central pressure pulse profiles collected with a catheter in a patient with left ventricular failure (#2) and normal heart function (#6).** Due to the shortened LVET the individual component pulses are clearly resolved in the case of patient #2.

Figure 16 presents the overlap of the central pulse pressure profiles, obtained with the catheter, of two patients, #2 and #6. Patient #2 was diagnosed with heart failure and ventricular dysfunction. 5 patients out of the cohort of patients displayed a similar central pulse pressure profile.

The pressure pulse profile obtained from patient #6 on the other hand corresponds to the “typical” central pulse pressure profiles [23]. In this case the left ventricular ejection time (LVET) is long enough that the returning renal (P2) and iliac reflection (P3) pulses overlap and merge. In this case the primary ejection pulse has a duration of about 310 milliseconds, as determined by measuring the time difference between pulse onset and dichrotic notch.

The pulse pressure profile of patient #2 indicates, on the other hand, a significantly shortened LVET. Based on the width of the primary feature in his central pressure profile, LVET is about 140 milliseconds. Since the primary ejection pulse (P1) is short in duration, so are its two reflection pulses P2 and P3, which feature the same temporal width. Furthermore, because ejection ceases before the reflections return, in contrast to patient #6, the three primary component pulses are clearly resolved.

**Figure 17:**
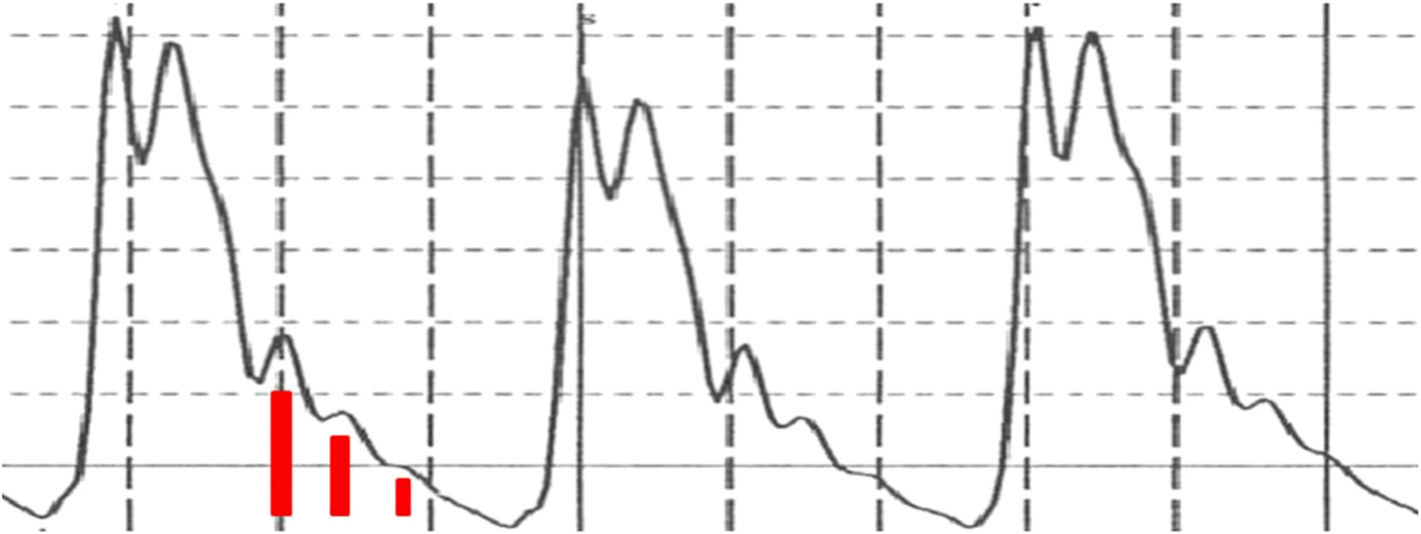
**Central catheter pressure pulse profile for patient 38, where all of the component pulses (P1 through P5) are clearly resolved.** Red vertical bars indicate positions of, respectively, P3, P4, P5.

The resolution varied at which the component pulses due to re-reflections, P4 and P5, could be observed in the tail end of the central pulse pressure profile. Figure 17 presents an example of data, for patient 38, where these component pulses are clearly resolved. The spectra of 35 patients displayed similar tail structure. As another example, the tail end of the patient #2 pulse shape in Figure 16 displays the next component pulse (P4) past the iliac pulse (#3), which is visible at the P2-P3 spacing. The tail end of the pulse profile of patient #6, on the other hand, displays no further tail structure.

## Discussion

The quantitative agreement between central line pressures and blood pressures derived using the PDA formalism within AAMI SP10 guidelines is one of the significant findings of this study. While the observation period per patient of 90 seconds was short, it frequently included episodes of significant dynamic changes in blood pressure, an aspect that is not part of the standard and that underlines the significance of the agreement of the two methodologies.

The results also strongly support the physical picture proposed by the PDA model. For the first time the primary three component pulses, P1, P2, and P3, as well as the harmonics P4 and P5, all of which were previously observed only either by indirect means [21] or by the CareTaker technology that is still being vetted, were observed directly using an accepted Gold Standard.

The pulse spectra of patient #2 presented in Figure 16 are the physiological equivalent of the table-top liquid injection experiments into distensible tubes performed by O’Rourke [24]. As part of those experiments constant-duration liquid pulses were injected into a distensible tube while an occlusion, either complete or incomplete, at the other end of the tube was moved closer and closer to the point of injection. With the occlusion removed from the point of injection by a distance longer than the product of pulse duration and tube pulse propagation velocity a distinct sequence of pulses is observed, the first being the injection pulse while the following pulses are reflections and re-reflections. As the occlusion end is moved closer to the injection end, the pulses merge into an envelope whose tail is offset from the envelope foot, or not, depending on whether the occlusion is close to complete or only partial.

The physical situation of the reflecting central pressure pulse in patient 2, with shortened ejection time, and 6, with normal ejection time, is equivalent. Focusing initially on the interaction between the primary ejection pulse and its interaction with the renal reflection site, it is patient 2’s short ejection time, relative to the time it takes the pulse to travel the distance to the renal reflection site and back, that ends the primary pulse before the renal reflection (P2) returns. As a result the two pulses are distinctly resolved, a condition that under healthy conditions is not the case.

The physical pulse reflection scenario extends readily to the iliac reflection site, which gives rise to the P3 component pulse. Of the same temporal extent as the ejection pulse P1 that gave rise to it, it arrives with a delay relative to the P2 pulse that is proportional to the arterial path corresponding to the abdominal artery to the iliac bifurcation, traveled in both directions.

Of interest in this context is also the question why the P2 component pulse amplitude is larger than the P3 component pulse amplitude, even though the iliac reflection site exhibits, at resting blood pressures, a much larger reflection coefficient than the renal reflection site (30-40% versus about 17%) [19]. The explanation is related to the changes in relative pulse foot/pulse tail baseline offsets observed by O’Rourke, [20] where, with increasing occlusion, the decay time of the end section of the pressure pulse increases. The physiological equivalent in the observations reported here is peripheral arterial resistance, which is critical to maintain pressure, albeit decaying, in the aorta during diastole. The effect on the component pressure pulses is to elongate them on their tail end, providing a pressure amplitude offset on top of which the pressure amplitude of the next arriving (reflection) component pulse is added. This effect is most pronounced for the P2 pulse which, as a result, is elevated to a pressure amplitude higher than that of the P1 pulse due to the P1 pulse’s decaying tail end.

Of interest also is the question why the harmonic component pulses P4 and P5 in the tail section of the central pressure pulse profile were observed in some patients and not in others. This is likely due to two physical factors. 1. Normal ejection times on the order of 300 milliseconds will tend to broaden out the harmonic reflections and cause them to overlap, diminishing their observability. 2. Both reflection sites, and in particular the iliac reflection site, have to be appreciably reflective to give rise to harmonic reflections. But this may not be case in the physiologically challenged and older cohort of patients that were part of this study. A fact that has been observed is that the reflection coefficient of the iliac reflection site diminishes with age, [25] a physiologically detrimental effect that leads to a lowered pressure amplitude outside the closed aortic valve during diastole, with commensurately lower perfusion of the coronary arteries.

The distributions of the slopes of the linear correlations (Figures 14 and 15) provide insight in regard to the relative arterial compliance of this cohort. Particularly interesting is a comparison of the distributions observed here with the average and the standard deviation of these slopes, or gain factors, observed in a significantly younger (average age: 24.4 years, SD: 3.0 years) and healthier 16-subject cohort (no diagnosed conditions) that were part of a lower-body-negative-pressure study [17]. The blue marker in Figure 11 (at 102.4 ± 23.2) and Figure 12 (at 0.19 ± .015) provides the graphical comparison. The distributions of the gain factors of this study have higher averages and are skewed toward the higher-value side, suggesting diminished arterial wall elasticity, which is a plausible expectation, given the age and cardiovascularly challenged state of the study population.

A reasonable question in this context is how, in light of potentially widely diverging arterial compliances, the conversion from PDA parameters to blood pressures will be accomplished, as it will be necessary for a general patient health monitor. A general response is that significantly more information beyond blood pressure trends resides in the arterial pressure pulse envelope. More specifically, arterial compliance has a distinct and differential effect on the temporal response of some of the component pressure pulses, providing an opportunity to isolate the effect of compliance and, even more importantly, of compliance changes. The implementation of these considerations is the object of ongoing work, the results of which will be published shortly.

The quantitative agreement between central line pressures and blood pressures derived from the PDA parameters supports the hypothesized physical picture. In the case of the P2P1 parameter the renal reflection (P2 pulse) originates at the junction between thoracic and abdominal aorta, a junction that is characterized by a significant change in arterial diameter. Since the thoracic aorta is the softest artery in the body, as evidenced by the fact that it exhibits the lowest pulse pressure propagation velocities (4–5 m/s), and is significantly more extensible than the abdominal aorta, increasing peak pressure, or systole, will enlarge the diameter mismatch, giving rise to a more pronounced renal reflection pulse amplitude while falling systole will produce the opposite effect, an effect observed in manipulative experiments performed by Latham [19]. The critical insight then is that the amplitude of the renal reflection will increase relative to the amplitude of the primary systolic (P1 pulse) peak because, while both component pulses travel the arteries of the arm complex, and are therefore both subject to the pulse narrowing and heightening due to the taper and wall composition changes of the peripheral arteries, only the renal reflection will have sampled the pressure-induced aortic impedance mismatch changes. This establishes the motivation for taking the ratio of the amplitudes of the #2 and the #1 pulse, which is P2P1.

A similarly physical argument can be made for the difference in arrival times of the primary pulse (#1) and the iliac reflection (#3), or T13. The difference in the arrival times of the primary arterial pulse, that is the left ventricular ejection, and the iliac reflection pulse is determined by the differential velocities with which both pulses propagated along their arterial paths. In the case of the iliac reflection the path length is longer than that of the primary pulse by almost twice the length of the torso. More importantly, both pulses travel at different velocities because their pressure amplitudes are different. Specifically, the iliac reflection pulse amplitude, which is determined by the reflection coefficient of the iliac reflection site, is on the order of 40% of pulse pressure. Both pulses therefore load the arterial wall differently during their arterial travel, as a result of which their propagation velocities are different. This point was validated in the previously mentioned LBNP experiments [17]. The second insight is that, because the pressure/velocity response curve is non-linear, a result known since the 1960s and based on Anliker’s work, [26] both pulses accelerate and decelerate at different rates as the pressure rises and falls. The primary pulse experiences the highest changes in velocity as a function of changes in blood pressure because it is subject to the steepest section of the pressure/velocity response curve, while the iliac pulse, “running” at much lower pressure, changes velocity much more gradually. Changes in the time of arrival therefore then reflect changes in the differential arterial pressure that the two pulses experience. While this differential pressure is not exactly pulse pressure, that is the difference between the full pulse arterial pulse height and the diastolic pressure floor, it represents about 60%-70% of it. Consequently the question arises how useful the differential pressure, which is reflected in the time interval T13, is in approximating pulse pressure. The response is that T13 corresponds to the pressure amplitude difference between the primary ejection pulse (#1) and the iliac reflection (#3). This amplitude difference covers the most important part of the pressure/velocity response curve, this being the exponential response section. The amplitude of the #3 pulse is entirely in the diastolic regime, which is linear, as Anliker showed [27]. However, it is the exponential section that is responsible for 95% of the dynamic pressure/velocity response characteristics, the rest being simply a linear offset.

Another point that the PDA model clarifies is the frequent mix-up between the dichrotic notch that is observed in central arterial pressure pulse profiles in the vicinity of the aortic valve and the inzisura that is observed in the pressure pulse profile in the arterial periphery. The mechanism giving rise to the dichrotic notch is well understood and accepted; as left ventricular pressure drops below aortic root pressure the flow reverses and the aortic valve slams shut, lowering the pressure momentarily in a notch-like manner in the aorta [28]. The notch, however, is only observed with manometers positioned within a few centimeters of the aortic valve. Latham’s data, for example, clearly shows the effect of the mechanical filtering, due to the arterial wall, that the sharp feature experiences as the monitoring site moves away. By the time the pressure pulse reaches the abdominal aorta, the feature is entirely gone [19,20]. Clearly then the inzisura observed in the arterial periphery is not the same feature, a fact underscored by the poor correlations obtained in experiments comparing the two [29]. Based on the PDA model the phenomenon instead arises out of the relative superposition of the three primary component pulses whose arrival time is delayed by the different arterial distances they traverse.

### Perspectives

We have presented supporting evidence for the physical model proposed by the Pulse Decomposition Analysis. The hypothesized components pressure pulses were observed directly using the Gold Standard methodology for central blood pressure measurements, a central line catheter, and the quantitative agreement between centrally measured blood pressures and PDA parameter-based blood pressures met AAMI SP-10 guidelines. It is hoped that the model’s comprehensiveness, simplicity, and physical basis will enhance the study of the human arterial pressure pulse.

## Competing interests

The authors declare that they have no competing interests.

## Authors’ contributions

MCB, KK and DWG conceived of the study, and participated in its design and coordination and helped to draft the manuscript. MCB performed the analysis with assistance from DWG and CMA. KK served as the PI of the study at the University of Virginia and assisted in the writing and editing of the manuscript. All authors read and approved the final manuscript.

## References

[B1] Martina JR(1) Westerhof BE van Goudoever J de Beaumont EM Truijen J Kim YS Immink RV Jöbsis DA Hollmann MW Lahpor JR de Mol BA van Lieshout JJ Noninvasive continuous arterial blood pressure monitoring with Nexfin® Anesthesiology 2012 116 5 1092 1103 22415387 10.1097/ALN.0b013e31824f94ed

[B2] Imholz BP(1) Wieling W van Montfrans GA Wesseling KH Fifteen years’ experience with finger arterial pressure monitoring: assessment of the technology Cardiovasc Res 1998 38 3 605 616 9747429 10.1016/s0008-6363(98)00067-4

[B3] Hager H(1) Mandadi G Pulley D Eagon JC Mascha E Nutter B Kurz A A comparison of noninvasive blood pressure measurement on the wrist with invasive arterial blood pressure monitoring in patients undergoing bariatric surgery Obes Surg 2009 19 6 717 724 18618207 10.1007/s11695-008-9607-7

[B4] Meidert AS(1) Huber W Müller JN Schöfthaler M Hapfelmeier A Langwieser N Wagner JY Eyer F Schmid RM Saugel B Radial artery applanation tonometry for continuous non-invasive arterial pressure monitoring in intensive care unit patients: comparison with invasively assessed radial arterial pressure Br J Anaesth 2014 112 3 521 528 24355832 10.1093/bja/aet400

[B5] Zorn EA(1) Wilson MB Angel JJ Zanella J Alpert BS Validation of an automated arterial tonometry monitor using Association for the Advancement of Medical Instrumentation standards Blood Press Monit 1997 2 4 185 188 10234114

[B6] Ott C Haetinger S Schneider MP Pauschinger M Schmieder RE Comparison of two noninvasive devices for measurement of central systolic blood pressure with invasive measurement during cardiac catheterization J Clin Hypertens (Greenwich) 2012 14 9 575 579 22947354 10.1111/j.1751-7176.2012.00682.x PMC8108982

[B7] Dewhirst E(1) Corridore M Klamar J Beebe A Rice J Barry N Tobias JD Accuracy of the CNAP monitor, a noninvasive continuous blood pressure device, in providing beat-to-beat blood pressure readings in the prone position J Clin Anesth 2013 25 4 309 313 23685101 10.1016/j.jclinane.2013.01.010

[B8] Agarwal R Light RP The effect of measuring ambulatory blood pressure on nighttime sleep and daytime activity–implications for dipping Clin J Am Soc Nephrol 2010 5 2 281 285 20019118 10.2215/CJN.07011009 PMC2827604

[B9] Dolan E(1) Stanton A Thijs L Hinedi K Atkins N McClory S Den Hond E McCormack P Staessen JA O’Brien E Superiority of ambulatory over clinic blood pressure measurement in predicting mortality: the Dublin outcome study Hypertension 2005 46 1 156 161 15939805 10.1161/01.HYP.0000170138.56903.7a

[B10] Kim BK Kim YM Lee Y Lim YH Shin J A reverse dipping pattern predicts cardiovascular mortality in a clinical cohort J Korean Med Sci 2013 28 10 1468 1473 doi: 10.3346/jkms.2013.28.10.1468. Epub 2013 Sep 25 24133351 10.3346/jkms.2013.28.10.1468 PMC3792601

[B11] Egan BM Zhao Y Axon RN US trends in prevalence, awareness, treatment, and control of hypertension, 1988–2008 JAMA 2010 303 20 2043 20501926 10.1001/jama.2010.650

[B12] Williams B Lacy PS Yan P Hwee CN Liang C Ting CM Development and validation of a novel method to derive central aortic systolic pressure from the radial pressure waveform using an N-point moving average method J Am Coll Cardiol 2011 57 8 951 961 21329842 10.1016/j.jacc.2010.09.054

[B13] Adji A O'Rourke MF Determination of central aortic systolic and pulse pressure from the radial artery pressure waveform Blood Press Monit 2004 9 3 115 21 15199304 10.1097/01.mbp.0000132426.32886.e0

[B14] Miyashita H Clinical Assessment of Central Blood Pressure Curr Hypertens Rev 2012 8 2 80 90 22866025 10.2174/157340212800840708 PMC3409361

[B15] Takazawa K Kobayashi H Kojima I Aizawa A Kinoh M Sugo Y Shimizu M Miyawaki Y Tanaka N Yamashina A Avolio A Estimation of central aortic systolic pressure using late systolic inflection of radial artery pulse and its application to vasodilator therapy J Hypertens 2012 30 5 908 916 22469836 10.1097/HJH.0b013e3283524910

[B16] Norton GR Majane OH Maseko MJ Libhaber C Redelinghuys M Kruger D Veller M Sareli P Woodiwiss AJ Brachial blood pressure-independent relations between radial late systolic shoulder-derived aortic pressures and target organ changes Hypertension 2012 59 4 885 892 22331378 10.1161/HYPERTENSIONAHA.111.187062

[B17] Baruch MC Warburton DE Bredin SS Cote A Gerdt DW Adkins CM Pulse Decomposition Analysis of the digital arterial pulse during hemorrhage simulation Nonlinear Biomed Phys 2011 5 1 1 21226911 10.1186/1753-4631-5-1 PMC3025935

[B18] Kríz J Seba P Force plate monitoring of human hemodynamics Nonlinear Biomed Phys 2008 2 1 1 18294366 10.1186/1753-4631-2-1 PMC2315646

[B19] Latham RD Westerhof N Sipkema P Rubal BJ Reuderink P Murgo JP Regional wave travel and reflections along the human aorta: a study with six simultaneous micromanometric pressures Circulation 1985 72 1257 1269 4064270 10.1161/01.cir.72.6.1257

[B20] Nichols WW O’Rourke MF McDonald’s blood flow in arteries. Theoretical, experimental and clinical principles 1999 London: Edward Arnold

[B21] Kriz J Martinik K Seba P Tosnerova V Force Plate Measurement of Human Hemodynamics 2006 http://cdsweb.cern.ch/record/855869/files/0507135.pdf?version=1

[B22] Nichols WW O’Rourke MF McDonald’s blood flow in arteries. Theoretical, experimental and clinical principles 1999 London: Edward Arnold p. 469, figure 22.27 A

[B23] Nelson MR Stepanek J Cevette M Covalciuc M Hurst RT Tajik AJ Noninvasive measurement of central vascular pressures with arterial tonometry: clinical revival of the pulse pressure waveform? Mayo Clin Proc 2010 85 5 460 472 20435839 10.4065/mcp.2009.0336 PMC2861976

[B24] O’Rourke MF Yaginuma T Wave reflections and the arterial pulse Arch Intern Med 1984 144 2 366 371 6365010

[B25] Greenwald SE Carter AC Berry CL Effect of age on the in vitro reflection coefficient of the aortoiliac bifurcation in humans Circulation 1990 82 1 114 123 2364509 10.1161/01.cir.82.1.114

[B26] Anliker M Histand MB Ogden E Dispersion and Attenuation of Small Artificial Pressure Waves in the Canine Aorta Circ Res 1968 23 539 551 5677945 10.1161/01.res.23.4.539

[B27] Anliker M Moritz WE Ogden E Transmission characteristics of axial waves in blood vessels J Biomech 1968 1 4 235 246 16329428 10.1016/0021-9290(68)90019-5

[B28] Smith D Craig E Mechanism of the dicrotic pulse Br Heart J 1986 56 531 534 3801244 10.1136/hrt.56.6.531 PMC1216400

[B29] Fahrenberg J Foerster F Müller W Non-invasive estimations of ventricular ejection time and stroke volume: comparison of impedance cardiography and the Portapres 2 J Med Eng Technol 1997 21 1 15 22 9080357 10.3109/03091909709030298

